# Chemometric Analysis Based on GC-MS Chemical Profiles of Three *Stachys* Species from Uzbekistan and Their Biological Activity

**DOI:** 10.3390/plants11091215

**Published:** 2022-04-29

**Authors:** Haidy A. Gad, Elbek A. Mukhammadiev, Gokhan Zengen, Nawal M. Al Musayeib, Hidayat Hussain, Ismail Bin Ware, Mohamed L. Ashour, Nilufar Z. Mamadalieva

**Affiliations:** 1 Department of Pharmacognosy, Faculty of Pharmacy, Ain Shams University, Cairo 11566, Egypt; haidygad@pharma.asu.edu.eg; 2 Institute of the Chemistry of Plant Substances, Academy of Sciences of RUz, Mirzo Ulugbek Str. 77, Tashkent 100170, Uzbekistan; elbekmuhammadiyev93@gmail.com; 3 Department of Biology, Selcuk University, 42130 Konya, Turkey; gokhanzengin@selcuk.edu.tr; 4 Department of Pharmacognosy, College of Pharmacy, King Saud University, Riyadh 11495, Saudi Arabia; nalmusayeib@ksu.edu.sa; 5 Department of Bioorganic Chemistry, Leibniz Institute of Plant Biochemistry, Weinberg 3, D-06120 Halle, Germany; hidayat.hussain@ipb-halle.de (H.H.); ismailbin.ware@ipb-halle.de (I.B.W.); 6 Institute of Botany, Academy of Sciences of RUz, Durmon Yuli Str. 32, Tashkent 100125, Uzbekistan

**Keywords:** *Stachys*, GC-MS, volatile components, antioxidants, enzyme inhibition, chemometric analysis

## Abstract

The chemical composition of the essential oils (EOs) of *Stachys byzantina*, *S. hissarica* and *S. betoniciflora* growing in Uzbekistan were determined, and their antioxidant and enzyme inhibitory activity were assessed. A gas chromatography-mass spectrometry (GC-MS) analysis revealed the presence of 143 metabolites accounting for 70.34, 76.78 and 88.63% of the total identified components of *S. byzantina*, *S. hissarica* and *S. betoniciflora,* respectively. Octadecanal (9.37%) was the most predominant in *S. betoniciflora*. However, n-butyl octadecenoate (4.92%) was the major volatile in *S. byzantina.* Benzaldehyde (5.01%) was present at a higher percentage in *S. hissarica.* A chemometric analysis revealed the ability of volatile profiling to discriminate between the studied *Stachys* species. The principal component analysis plot displayed a clear diversity of *Stachys* species where the octadecanal and benzaldehyde were the main discriminating markers. The antioxidant activity was evaluated in vitro using 2,2-diphenyl-1-picryl-hydrazyl (DPPH), 2,2-azino bis (3-ethylbenzothiazoline-6-sulphonic acid) (ABTS), cupric reducing antioxidant capacity (CUPRAC), ferric reducing power (FRAP), chelating and phosphomolybdenum (PBD). Moreover, the ability of the essential oils to inhibit both acetyl/butyrylcholinesterases (AChE and BChE), α-amylase, α-glucosidase and tyrosinase was assessed. The volatiles from *S. hissarica* exhibited the highest activity in both the ABTS (226.48 ± 1.75 mg Trolox equivalent (TE)/g oil) and FRAP (109.55 ± 3.24 mg TE/g oil) assays. However, *S. betoniciflora* displayed the strongest activity in the other assays (174.94 ± 0.20 mg TE/g oil for CUPRAC, 60.11 ± 0.36 mg EDTA equivalent (EDTAE)/g oil for chelating and 28.24 ± 1.00 (mmol TE/g oil) for PBD. Regarding the enzyme inhibitory activity, *S. byzantina* demonstrated the strongest AChE (5.64 ± 0.04 mg galantamine equivalent (GALAE)/g oil) and tyrosinase inhibitory (101.07 ± 0.60 mg kojic acid equivalent (KAE)/g) activity. The highest activity for BChE (11.18 ± 0.19 mg GALAE/g oil), amylase inhibition (0.76 ± 0.02 mmol acarbose equivalent (ACAE)/g oil) and glucosidase inhibition (24.11 ± 0.06 mmol ACAE/g oil) was observed in *S. betoniciflora*. These results showed that EOs of *Stachys* species could be used as antioxidant, hypoglycemic and skincare agents.

## 1. Introduction

The genus *Stachys* L. is one of the largest genera in the family *Lamiaceae* (*Labiatae*) that includes about 300 species of herbaceous annuals and perennial plants in the subtropical and tropical regions of the world [1]. The species name comes from Greek and means ‘‘an ear of grain”, referring to the shape of the inflorescence spike that is present in many members [2].

*Stachys* plants (known as mountain tea) have traditionally been consumed in various countries as decoction or infusion teas. These extracts were used to treat various ailments, such as vaginal tumors, sclerosis of the spleen, asthma, cough, ulcers, abdominal pains, fever, diarrhea, sore mouth and throat, internal bleeding, and weaknesses of the liver and heart in addition to inflammatory and rheumatic disorders [3,4,5,6].

Many *Stachys* species are known as alimentary plants that are consumed for preparing foods such as yoghourt or jelly to improve the taste and flavor [7]. In Poland, various organs of *S. palustris* are used as soup and vodka additives [3]. The tubers can also be dried and crushed into a powder for making bread [8] due to their nutritional value as a rich source of carbohydrates, even for diabetic people, not only for humans but also for wild pigs [9,10,11].

The literature reported the pharmacological activities of different *Stachys* species as antimicrobial [6,12], anticancer [13,14], antioxidant [15,16,17], antitumor [6,18], antinociceptive and anti-inflammatory [19], with anti-Alzheimer, antidiabetic and anti-obesity potential [20] in addition to the treatment of anxiety [5].

The wide range of applications of various *Stachys* species, either as food or medicinal remedies, is mainly attributed to the major volatile components. Many studies reported the principal active metabolites present in the ethereal oil of different *Stachys* species worldwide [4]. Essential oils of the aerial parts of many *Stachys* species collected from Iran were determined, where the main identified components were linalool, α-terpineol, germacrene D, α/β-pinene and α/β-phellandrene [16,20,21,22,23]. Similar results were obtained for those species growing in Turkey [7,24]. In Greece, the major identified components were caryophyllene, caryophyllene oxide and α-calacorene, [25,26,27]. Comparable results were obtained by analyzing the *Stachys* essential oils in Serbia and Montenegro [28,29].

Regarding *S. byzantina* K. Koch., (syn. *Stachys lanata*) commonly known as “lamb’s ear” or “lamb’s tongue”, growing in the north and north-west of Iran, Turkey, Caucasia and Afghanistan [30]. Leaf infusions have traditionally been used as anti-inflammatories in Brazil [31]. However, leaf decoctions have been applied to infected wounds and cuts in Iran [32,33].

The genus *Stachys* is well-distributed in Uzbekistan [34]. *Stachys betoniciflora* Rupr and *Stachys hissarica* Rgl. are widely used as traditional remedies in Uzbekistan. *S. betoniciflora* has been used in traditional medicine for the treatment of diarrhea, genital tumors, hemorrhage, inflammation, heart disease, cough, ulcers, menstrual irregularities, bleeding, hysteria, hypertension, epilepsy, fainting, gout, jaundice and rheumatism [34]. A tea made from the herb is used to treat gastrointestinal pain, hemoptysis, respiratory disease, inflammation of the kidneys and bladder, and is also used as a sedative. An infusion of the roots is used as a laxative [35]. However, the folklore uses have never been scientifically evaluated, and other medicinal benefits of these plants are unknown.

The aim of this study was to investigate the essential oils of different *Stachys* species growing in Uzbekistan via GC-MS. *S. byzantina, S. hissarica* and *betoniciflora* were investigated, and this was the first such investigation for the two later species. In addition, the principal component analysis (PCA) and hierarchical cluster analysis (HCA) were applied to discriminate various *Stachys* species based on their chemical profiles rather than their growing location, which could be used as a tool for the proper use of the plants and prevent adulteration. Furthermore, the volatile oils obtained from different *Stachys* species were investigated for their antioxidant and enzyme inhibitory activities for the first time for these species.

## 2. Results and Discussion

### 2.1. Chemical Composition of Volatile Compounds

The chemical composition of the volatile oils of three fully grown *Stachys* species present in Uzbekistan, namely *S. byzantina*, *S. hissarica* and *S. betoniciflora*, were investigated via GC-MS analyses (Table 1). The yields of these species were 0.3, 0.04 and 0.03% *w*/*w*, respectively. The selection of spring was made to ensure the full maturity and maximum yields for the oils. The samples comprised 143 constituents that represented 70.34, 76.78 and 88.63% of the total identified compositions in *S. byzantina*, *S. hissarica* and *S. betoniciflora,* respectively. Octadecanal (9.37%), eucalyptol (6.89%), linalool (6.66%) and p-cymene (5.2%) were the most predominant components in *S. betoniciflora*. Eucalyptol (6.89%), α-thujene (1.44%), α-pinene (3.98%) and D-limonene (2.4%) were among the components that were only detected in *S. betoniciflora*. Regarding *S. hissarica*, benzaldehyde (5.01%), n-hexadecanoic acid (4.68%) and pulegone (4.68%) were the chief compounds detected. In addition, thymol (2.74%), 2-hexenal (2.61%), dihydroactinidiolide (2.27%), tetradecanoic acid (2.05%) and eugenol (2.05%) were present in *S. hissarica* at higher levels compared to the other species. However, n-butyl octadecenoate (4.92%) and n-triacontane (4.25%) displayed the maximum percentage in *S. byzantina*. Furthermore, cis-3-hexenol (3.71%), hexahydrofarnesyl acetone (3.63%), 9,12,15-octadecatrienoic acid, methyl ester (3.42%), n-hexadecanoic acid (3.16%) and (+)-δ-cadinene (3.13%) were among the constituents identified in *S. byzantina*.

Our results were close to those reported for the essential oil composition of the aerial parts of *S. byzanthina* in Iran, where α-copaene (16.5%), spathulenol (16.1%), β-caryophyllene (14.3%) and β- cubebene (12.6%) were the major components [36,37]. In another study, α-thujone (25.9%), α-humulene (24.9%), β-caryophyllene (12.6%) and viridiflorol (10.5%) were reported as main components [38]. On the contrary, the dried flowering aerial parts of *S. byzantina* oil showed a different pattern, where piperitenone (9.9%), 6,10,14-trimethyl pentadecan-2-one (6.4%) and n-tricosane (6.4%) were predominant [39]. However, germacrene D (9.6%), menthone (6.9%) and 1,8-cineole (14.8%) were the major identified components in *S. byzantina* collected from Urmia, Azerbaijan [40]. It is noteworthy to point out that the essential oil components can be affected by such factors as geographical, climatic, seasonal and experimental conditions. However, our main aim was not to assess the effect of these factors but to use the difference in discriminating the species themselves [16].

*S. byzantina* showed significant levels of antioxidants in addition to cholinesterase enzymes, lipase, tyrosinase and α-glucosidase inhibition [20]. In Brazil, the essential oil of *S. byzanthina* showed strong antimicrobial activity against *Candida albicans,* where γ-muurolene (65%), valeranone (15.42%), β-elemene (6.83%) and cis-β-ocimene (1.39%) were the main components responsible for the antifungal activity [31]. In another study, hexahydrofarnesyl acetone (20.41%) was the main compound in *S. byzantina* with antimicrobial and antioxidant properties [17]. However, no previous studies reported the major active components present in *S. betoniciflora* and *S. hissarica.*

### 2.2. Chemometric Analysis Based on GC-MS Analysis

**Figure 1:**
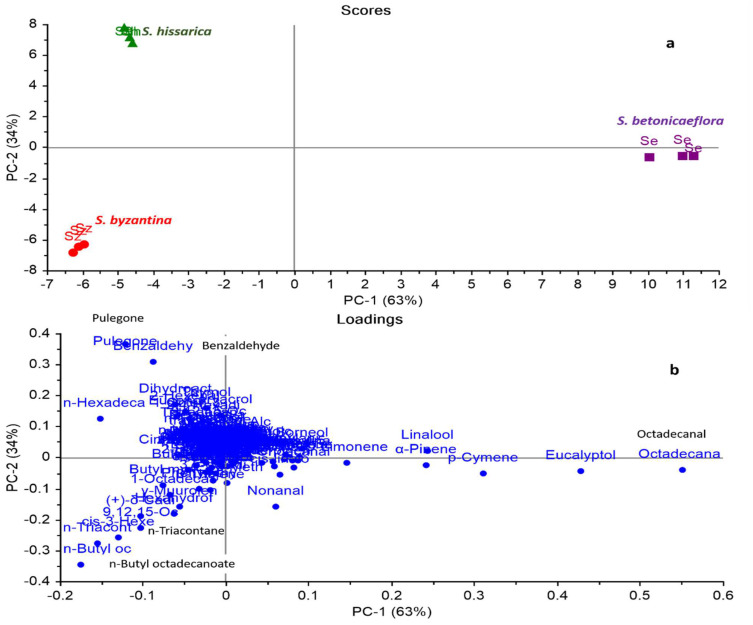
Principal component analysis score plot (**a**) and loading plot (**b**) of GC-MS aromatic profiles of different *Stachys* species, based on the identification of compounds shown in Table 1. *S. hissarica (Sh)*, *S. betoniciflora (Se)* and *S. byzantia (Sz)*.

Due to the complexity of the GC-MS-based data comprising the qualitative and quantitative discrepancies of various *Stachys* species, the multivariate analysis was applied using a principal component analysis (PCA) and a hierarchal cluster analysis (HCA) to discriminate between closely related *Stachys* species as well as to detect any significant relationship between these species. A matrix of all samples and their replicates (9 samples) multiplied by 143 variables (GC/MS peak area %) was constructed in MS Excel^®^, then subjected to a multivariate analysis (PCA and HCA). Owing to the large number of variables, PCA was applied to reduce the dimensionality of the multiple data sets in addition to removing the redundancy in the variables, where the 1st PC accounts for a maximal amount of total variable variance in the observed data, and the 2nd PC accounts for a maximal amount of variance in the data set that was not accounted for by the first component. No transformation was performed, and the model was constructed utilizing raw data (peak area % for each compound as in Table 1). Figure 1a,b represent the PCA score and loading plots based on the GC-MS chemical profiles of the volatile components in the three *Stachys* species, respectively.

The result of this study indicated that there is a significant variance among the different *Stachys* species. The first two principal components explained about 97% of the variation in the dataset, with PC1 accounting for 63% and PC2 accounting for 34% of the variance. The three *Stachys* samples were clearly dispersed along the PCs in the PCA plot. *S. byzantina* and *S. hissarica*, in particular, exhibited negative PC1 scores and were positioned on the left side of the figure, while *S. betoniciflora* was plotted on the other side with a positive PC1 score.

Within the most discriminating compounds on positive PC1, octadecanal and eucalyptol were identified in *S. betoniciflora.* However, n-butyl octadecenoate and n-triacontane were the main differentiating markers in *S.* *byzantina.* Nevertheless, benzaldehyde (5.01%), n-hexadecanoic acid (4.68%) and pulegone were the major distinctive bioactive compounds discriminating *S. hissarica.*

**Figure 2:**
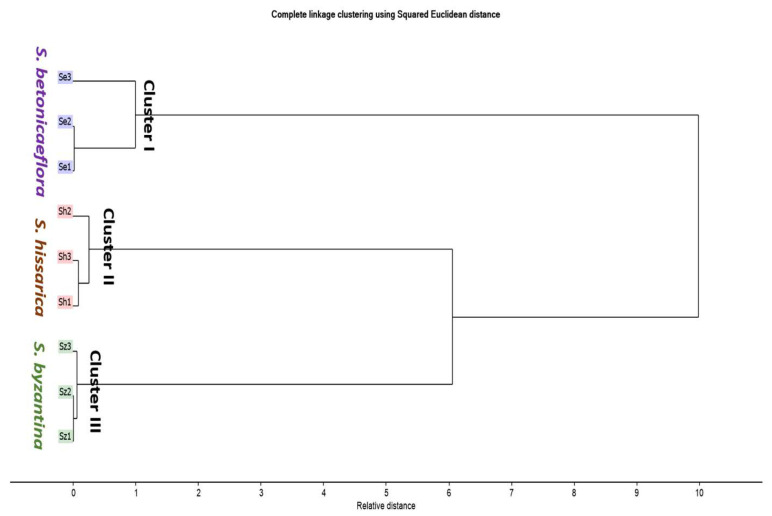
HCA dendrogram of different *Stachys* species based on the identification of compounds shown in Table 1. *S. hissarica (Sh), S. betoniciflora (Se)* and *S. byzantina (Sz)*.

An HCA clustering was performed to have a better insight into species classification. Figure 2 shows the HCA dendrogram based on the GC-MS aromatic profiles. The HCA dendrogram clustered the *Stachys* species into three main clusters. Clusters I, II and III displayed S. *betoniciflora*, *S. byzantina* and *S. hissarica,* respectively. The dendrogram confirmed the results obtained from the PCA, revealing the closeness of *S. byzantina* and *S. hissarica*.

### 2.3. Antioxidant Activity of Stachys Species

The antioxidant potential of the essential oil (EO) samples was assessed in vitro according to various assays comprising the 2,2-diphenyl-1-picryl-hydrazyl (DPPH), 2,2-azino bis (3-ethylbenzothiazoline-6-sulphonic acid) (ABTS), cupric reducing antioxidant capacity (CUPRAC), ferric reducing power (FRAP), chelating and phosphomolybdenum (PBD) assays. The results are presented in the form of Trolox equivalent (TE) and ethylenediaminetetraacetic acid equivalent (EDTAE).

The results displayed in Table 2 revealed that the studied samples showed moderate antioxidant potential in the assays. Most of the species showed antioxidant potential in the performed assays, except for the DPPH assay where only *S. hissarica* exhibited activity (13.04 ± 0.97 mg TE/g oil). Concerning the ABTS and FRAP assays, *S. hissarica* displayed the best activity (226.48 ± 1.75 and 109.55 ± 3.24 mg TE/g oil), followed by *S. betoniciflora* (128.54 ± 1.32 mg and 56.94 ± 0.75 mg TE/g oil), respectively. On the contrary, *S. betoniciflora* displayed the strongest activity in the other assays (174.94 ± 0.20 mg TE/g oil for CUPRAC, 60.11 ± 0.36 mg EDTAE/g oil for chelating and 28.24 ± 1.00 mmol TE/g oil for PBD), followed by *S. hissarica* (162.62 ± 1.15 mg TE/g oil for CUPRAC, 24.30 ± 1.50 mg EDTAE/g oil for chelating and 5.69 ± 0.21 mmol TE/g oil for PBD. It was observed that *S. byzantina* exhibited the lowest antioxidant activity in all applied experiments. The obtained results could be explained by the chemical components of the tested essential oils. For example, the antioxidant properties of *S. hissarica* essential oil could be attributed to the presence of pulegone. Consistent with our observation, Torres-Martínez et al. [41] reported the significant antioxidant properties of pulegone. In addition, the presence of eucalyptol, linalool and *p*-cymene was linked to the antioxidant properties of *S. betoniciflora*. In previous studies [42,43,44], these compounds were described as remarkable radical scavengers and reductive agents. However, because higher levels of hydrocarbons (such as n-butyl octadecenoate and n-triacontane) were present in *S. byzantina* essential oil, it exhibited the weakest antioxidant properties.

Concerning *S. byzantina*, few studies reported the oxidation inhibition properties of its essential oil by various assays. *S. byzantina* exhibited a slight DPPH scavenging potential, with the value of 1.03 mg TEs/g EO. For the phosphomolybdenum test, it displayed 1.96 mmol TEs/g EO. Concerning the CUPRAC and FRAP assays, *S. byzantina* showed a high reducing power potential (81.79 and 44.77 mg TEs/g EO, respectively). In the chelating experiment, it revealed 15.65 mg EDTAEs/g EO [20].

### 2.4. Enzyme Inhibitory Activity of Stachys Species

The enzyme inhibitory potentials of the *Stachys* essential oils were assessed against various enzymes, including acetylcholinesterase (AChE), butyrylcholinesterase (BChE), tyrosinase, amylase and glucosidase. The enzymes were chosen to assess the potential of the tested essential oils in global health problems, the prevalence of which is increasing by the day. For example, cholinesterases are the main target in the treatment of Alzheimer’s disease. In addition, amylase and glucosidase are the major players in controlling blood sugar levels in diabetics. The results (Table 3) are presented in the form of galantamine equivalent (GALAE), kojic acid equivalent (KAE) and acarbose equivalent (ACAE).

In this study, *Stachys* EOs exerted a valuable cholinesterase inhibitory potential. The best AChE inhibitory potential was found in *S. byzantina* with 5.64 mg GALAE/g oil, followed by *S. hissarica* (5.05 mg GALAE/g oil) and *S. betoniciflora* (4.09 mg GALAE/g oil). However, the order for BChE was *S. betoniciflora* (13.81 mg GALAE/g oil) > *S. hissarica* (11.82 mg GALAE/g oil) > *S. byzantina* (11.18 mg GALAE/g oil). Nevertheless, our results were in accordance with those reported for *S. byzantina*, where the enzyme inhibition potential of *S. byzantina* essential oil was investigated to be 5.06 and 6.62 mg GALAEs/g EO against BChE and AChE, respectively [20]. Concerning tyrosinase inhibition, *S. byzantina* exhibited the strongest activity (101. 07 mg KAE/g oil), followed by *S. hissarica* (92.26 mg KAE/g oil) and *S. betoniciflora* (75.77 mg KAE/g oil). Concerning tyrosinase, *S. byzantina* EOs exhibited lower potency compared to our results, with 25.26 mg KAEs/g EO [20]. With regards to α-amylase activity, the highest and lowest activity were associated with *S. betoniciflora* and *S. byzantina* with values 0.76 and 0.59 mmol ACAE/g oil, respectively. Meanwhile, *S. hissarica* displayed an intermediate value (0.68 mmol ACAE/g oil). On the other side, the measurement of the α-glucosidase inhibition activity of the tested oils revealed that the strongest effect was presented by *S. betoniciflora* (24.89 mmol ACAE/g oil) compared to other *Stachys* species. With reference to α-amylase activity, *S. byzantina* displayed an approximately similar value with 0.74 mmol ACEs/g EO. On the other hand, the measurement of the α-glucosidase inhibition activity of the EOs showed *S. byzantina* with 4.56 mmol ACEs/g EO [20]. Similar to the antioxidant properties, the enzyme inhibitory properties can be linked to the chemical components of the tested essential oils. For example, Aazza et al. [45] reported that the cholinesterase inhibition abilities of several volatile compounds, including eucalyptol, which is found in *S. betoniciflora*. In addition, Basak and Ferda [46] reported the amylase and glucosidase inhibitory properties of eucalyptol. In a study performed by Matailo et al. [47], the BChE inhibitory effect of pulegone was described. However, we must take into account the presence of other compounds, even at low concentrations, because the essential oils are complex mixtures, and their constituents may exhibit different synergetic or antagonistic interactions.

## 3. Materials and Methods

### 3.1. Plant Material

Aerial parts of *Stachys byzantina* K. Koch (syn. *Stachys lanata* Jacq.) (N2016017) were collected from the botanical field of the Institute of the Chemistry of Plant Substances (Near Tashkent, Tashkent region, Uzbekistan). *Stachys hissarica* Regel (N2016023) was collected from Chimgan (Tashkent region), while *Stachys betoniciflora* Rupr. ex O. Fedtsch. et B. Fedtsch. (Syn. *Betonica betoniciflora* Rupr. ex O. Fedtsch. and B. Fedtsch. Sennikov) (N2016030) was collected from Aksay (Near Tashkent, Pskem Mountain Range, Uzbekistan). All the plants were collected in the fully grown stage during spring to ensure the maximum yields. The plants were identified by Dr. Olim Nigmatullaev and the voucher samples were deposited at the Herbarium of the Institute of Chemistry of Plant Substances, Academy of Sciences of Uzbekistan.

### 3.2. Essential Oil Isolation

The plant samples were air-dried at a temperature not exceeding 30 °C and then powdered before use. The preparation of the volatile samples of the powdered *Stachys* (100 g of powder in 1 L of distilled water) was performed by hydrodistillation for 2 h using a Clevenger-type apparatus. The oil samples were dried by passing over anhydrous sodium sulphate to remove the moisture and were maintained at 30 °C in dark brown and air-tight closed vials until their analyses.

### 3.3. GC-MS Analysis

The GC-MS characterization of volatile components was performed on an Agilent 7890 B gas chromatograph (Agilent Technologies, Rotterdam, The Netherlands) equipped with a VF-Wax CP 9205 fused silica column (100% polyethylene glycol, 30 m × 0.25 mm, 0.25 µm). It was coupled with a 5977A mass selective detector (Agilent Technologies). The interface temperature: 280 °C; MS source temperature: 230 °C; ionization energy: 70 eV; scan range: 45–950 atomic mass units. The sample (0.5 μL) was automatically injected into the chromatograph using a GC auto sampler. The oven temperature was kept at 50 °C for 5 min, then rising from 50 °C to 280 °C at 5 °C/min, and was finally held isothermally at 280 °C for 15 min; injector temperature 250 °C; detector temperature 270 °C; carrier gas helium (0.9 mL/min); with split mode (split ratio, 1:20). C7–C40 standard saturated alkanes were purchased from Sigma-Aldrich (Merck, Germany). Enhanced ChemStation software, version MSD F.01.01.2317 (Agilent Technologies) was used for recording and integrating the chromatograms. The compounds were identified by comparison of their mass-spectral data and retention indices (RIs) with those of the Wiley Registry of Mass Spectral Data (9th Ed.), NIST Mass Spectral Library (2011), references [48,49] and our own laboratory database [50].

### 3.4. Antioxidant and Enzyme Inhibitory Assays

Different procedures were applied to investigate the antioxidant activities of the tested Stachys oils. These procedures included free radical scavenging (DPPH and ABTS), reducing power (CUPRAC and FRAP), metal chelating and phosphomolybdenum. The experimental details were as previously described [51,52]. A similar method was applied for the inhibitory effects of the Stachys oils that were tested against different enzymes (tyrosinase, amylase, glucosidase and cholinesterase) [52,53]. Both the antioxidant and enzyme inhibition assays were described by standard equivalents (Trolox and EDTA for antioxidant, galantamine for cholinesterase, kojic acid for tyrosinase, and acarbose for amylase and glucosidase activity). All samples were analyzed in triplicate in three independent experiments.

### 3.5. Statistical Analysis

Unless otherwise specified in the technique, all tests were repeated three times. Continuous variables were expressed as means ± SD. A one-way analysis of variance (ANOVA) was used to determine statistical significance, followed by a multiple comparison test (Tukey’s post hoc test) with a significance level of *p* < 0.05.

### 3.6. Multivariate Analysis

The data obtained from the GC-MS were subjected to a chemometric analysis. A principal component analysis (PCA) was applied as an initiative phase in the data investigation to afford an overview of all sample groupings and to identify the markers responsible for this grouping. A hierarchal cluster analysis (HCA) was used to allow the clustering of samples. Hierarchical clustering techniques are based on the creation of branched structures, called dendrograms, which are qualitative in nature and permit the visualization of clusters and correlations amongst samples [54]. The clustering patterns were built by applying the complete linkage method. This exhibition is more effective when the distance between the samples (points) is computed by the squared Euclidean method. PCA and HCA were accomplished using CAMO’s Unscrambler^®^ X 10.4 software (Computer-Aided Modeling, AS, Norway).

## 4. Conclusions

*Stachys* species are used as pleasant and fragrant medicinal herbal teas due to their volatiles and bioactive principles. In the present study, a comparative investigation of the EOs obtained from three popular *Stachys* species, namely, *S. byzantina*, *S. hissarica* and *S. betoniciflora,* indicated that they showed modest natural antioxidant agents. Their major bioactive components were identified as octadecanal, eucalyptol, linalool, n-butyl octadecenoate, n-triacontane, benzaldehyde, n-hexadecanoic acid and pulegone. In addition, the EOs exhibited moderate enzyme inhibitory activities, demonstrating the health benefits of various *Stachys* species, such as neuroprotective, hypoglycemic and fat adsorption preventive effects. Accordingly, these EOs could be considered as promising candidates that could be used for many therapeutic conditions. However, more clinical trials are needed to validate their potential uses. This is the first report comparing *S. hissarica* and *S. betoniciflora* to *S. byzantina* growing in Uzbekistan utilizing a multivariate analysis based on GC-MS chemical profiles in addition to reporting their antioxidant and enzyme inhibitory activities.

## Figures and Tables

**Table 1:** Chemical composition of the volatile oils of the aerial parts of three *Stachys* species growing in Uzbekistan, namely *Stachys byzantina*, *S. hissarica* and *S. betoniciflora* (*n* = 3).

No.	Rt.	Compound	*m*/*z* Values	Retention Indix		Peak Area (%)		
				Cal.	Rep.	*S. Betoniciflora*	*S.* *Byzantina*	*S.* *Hissarica*
1.	6.45	α-Thujene *	136.23	1023	1025	1.44	-	-
2.	7.67	α-Pinene *	136.23	1031	1030	3.98	-	-
3.	8.23	β-Thujene *	136.23	1130	1133	0.54	1.49	
4.	8.4	β-Pinene *	136.23	1134	1137	0.42	-	-
5.	8.54	3-Carene *	136.23	1148	1149	0.47	-	-
6.	9.14	D-Limonene *	136.23	1201	1203	2.40	-	-
7.	9.54	Eucalyptol	154.24	1207	1208	6.89	-	-
8.	9.75	2-Hexenal *	98.14	1215	1216	0.74	0.68	2.61
9.	10.34	β-*cis*-Ocimene	136.23	1224	1225	0.19	-	-
10.	10.59	γ-Terpinene	136.23	1230	1232	1.14	0.32	
11.	10.89	β-*trans*-Ocimene	136.23	1238	1240	0.51	-	-
12.	11.39	*p*-Cymene *	134.22	1267	1268	5.20	0.25	-
13.	11.7	2-methyl-,2-methylbutyl ester Butanoic acid	172.26	1274	1276	1.33	-	-
14.	11.91	*n*-Octanal	128.21	1285	1287	0.18	-	-
15.	12.17	iso-Amyl isovalerate	172.26	1292	1294	1.17	-	-
16.	12.26	1-Octen-3-one	126.19	1296	1298	-	-	1.66
17.	12.64	2,4-Nonadienal	138.20	1304	1305	0.49	-	-
18.	12.74	3-Hexen-1-ol, acetate	142.19	1311	1313	0.21	0.39	-
19.	12.83	3-Methyl-cyclohexanone	112.16	1330	1333	-	-	0.16
20.	13.33	6-Methyl-5-hepten-2-one	126.19	1340	1342	1.34	-	0.14
21.	13.82	1-Hexanol	102.17	1357	1359	-	0.70	-
22.	14.4	*Allo*-Ocimene	136.23	1370	1372	0.82	0.18	-
23.	14.69	*cis*-3-Hexenol	100.15	1378	1379	-	3.71	-
24.	14.92	Nonanal	142.23	1390	1390	2.26	2.09	-
25.	15.08	2,4-Hexadienal	96.12	1394	1395	0.22	-	1.60
26.	15.28	(*E*)-2-Hexen-1-ol	100.15	1401	1402	-	0.48	-
27.	15.54	Butyl hexanoate	172.26	1413	1414	0.24	-	0.26
28.	15.83	*trans*-2-Octenal	126.19	1427	1427	0.21	-	0.15
29.	16.06	*p*-Cymenene *	132.20	1434	1435	0.51	-	-
30.	16.19	*cis*-Linalool oxide	170.24	1445	1445	1.80	0.99	0.35
31.	16.47	1-Octen-3-ol	128.21	1452	1453	-	0.47	0.69
32.	16.61	1-Heptanol	116.20	1455	1456	0.74	0.34	0.40
33.	16.96	*trans*-Linalool oxide	170.24	1464	1466	1.55	-	0.14
34.	17.4	*trans-p*-Menthan-3-one	154.24	1487	1486	-	-	0.86
35.	17.51	(*E,E*)-2,4-Heptadienal	110.15	1493	1493	1.31	0.43	0.31
36.	17.71	*n*-Decanal	156.26	1498	1498	-	0.10	-
37.	17.99	Camphor *	152.23	1506	1505	0.63	0.12	0.29
38.	18.24	Benzaldehyde *	106.12	1521	1520	1.31	0.99	5.01
39.	18.52	(*Z*)-2-Nonenal	140.22	1532	1532	0.18	-	0.13
40.	18.77	Pinocamphone	152.23	1565	1566	0.23	-	0.09
41.	18.96	Linalool *	154.24	1553	1554	6.66	0.45	1.04
42.	19.47	3,5-Octadiene-2-one	124.18	1568	1569	0.57	1.20	0.25
43.	19.79	*Iso* pulegone	152.23	1573	1574	-	-	0.40
44.	19.84	Bornyl acetate *	196.28	1579	1579	0.11	0.09	0.09
45.	19.97	(*E*)-6-Methyl-3,5-heptadien-2-one	124.18	1581	1582	0.65	1.09	-
46.	20.08	*trans*-Caryophyllene	204.35	1588	1589	0.29	0.10	0.28
47.	20.23	4-Terpineol *	154.24	1599	1598	0.59	0.24	0.83
48.	20.34	Undecanal	170.29	1603	1604	1.55	0.34	-
49.	20.43	Aromadendrene *	204.35	1608	1608	0.22	-	0.24
50.	20.52	Butyl octanoate	200.31	1617	1619	0.40	-	-
51.	20.66	β-Cyclocitral	152.23	1623	1624	0.19	-	0.35
52.	20.8	1-Terpineol	154.24	1629	1628	-	-	0.11
53.	20.87	Myrtenal *	150.21	1630	1632	0.28	0.22	0.13
54.	21.36	Pulegone *	152.23	1643	1644	0.11	-	4.68
55.	21.62	1-Nonanol	144.25	1655	1656	0.2	0.52	0.31
56.	21.7	Germacrene D	204.35	1676	1678	0.48	0.17	0.41
57.	21.82	α-Humulene	204.35	1680	1681	0.26	0.37	-
58.	21.95	4-Vinylanisole	134.17	1672	1670	1.28	-	0.25
59.	22.19	Neral *	152.23	1677	1679	0.26	-	-
60.	22.27	δ-Terpineol	154.24	1684	1684	0.20	-	0.67
61.	22.33	γ-Muurolene	204.35	1702	1703	0.38	2.23	-
62.	22.44	α-Terpineol	154.24	1711	1712	-	0.42	0.68
63.	22.55	Borneol *	154.24	1714	1715	1.74	-	0.52
64.	22.62	Verbenone	150.21	1722	1723	0.26	-	-
65.	22.78	Isoborneol	154.24	1695	1696	0.23	0.74	-
66.	22.83	Dodecanal	184.31	1720	1722	0.13	0.23	0.14
67.	22.92	Butyl nonanoate	214.34	1723	1725	0.15	1.19	0.56
68.	23.12	Piperitone *	152.23	1728	1729	0.62	0.59	0.44
69.	23.21	β-Bisabolene	204.35	1733	1733	0.43	0.26	0.26
70.	23.54	Naphthalene, 1,2-dihydro-1,1,6-trimethyl-	172.26	1748	1748	0.14	0.19	0.12
71.	23.59	Epoxylinalool	170,24	1750	1750	0.27	-	-
72.	23.86	(+)-δ-Cadinene	204.35	1752	1752	0.31	3.13	0.40
73.	23.99	1-Decanol	158.28	1761	1763	0.13	-	0.99
74.	24.33	*p*-Cumic aldehyde	148.20	1782	1783	0.35	0.22	0.31
75.	24.68	α-Cadinene	204.35	1784	1785	0.07	0.69	-
76.	24.83	*trans-p*-Mentha-1(7),8-dien-2-ol	152.23	1792	1793	0.11	-	-
77.	24.93	2,4-Decadienal	152.23	1796	1797	0.24	-	0.55
78.	25.14	β-Damascenone	190.28	1814	1815	0.36	0.14	0.16
79.	25.2	β-Damascone	192.29	1822	1824	-	0.36	0.52
80.	25.48	*trans*-Calamenene	202.33	1844	1845	0.28	0.26	-
81.	25.74	Geraniol *	154.24	1855	1856	0.31	-	0.24
82.	25.85	*n*-Hexanoic acid	116.15	1860	1861	0.78	-	0.49
83.	26	(*Z*)-Geranyl acetone	194.31	1867	1868	0.13	-	0.53
84.	26.32	*exo*-2-Hydroxycineole	170.24	1869	1870	0.68	0.17	0.24
85.	26.57	Benzyl alcohol	108.13	1884	1885	0.57	-	1.13
86.	26.89	(*E*)-2-Dodecenal	182.30	1887	1887	-	0.51	0.36
87.	27.22	α-Calacorene	200.31	1915	1916	0.18	0.33	0.82
88.	27.43	Tetradecanal	212.37	1920	1921	0.11	-	-
89.	27.56	Phenylethyl alcohol	122.16	1926	1927	0.33	1.45	-
90.	27.68	*trans*-β-Ionone	192.29	1930	1931	0.37	0.40	0.87
91.	27.73	*cis*-Jasmone	164.24	1937	1938	-	-	0.43
92.	27.9	2,6-Dimethyl-3,7-octadiene-2,6-diol	170.24	1945	1945	0.27	0.49	0.15
93.	28.02	2-Ethyl-hexanoic acid	144.21	1954	1954	0.32	1.05	0.66
94.	28.55	*cis*-Caryophyllene oxide *	220.35	1953	1955	0.38	0.95	0.36
95.	28.76	β-Ionone epoxide	208.29	1966	1967	0.38	0.31	0.79
96.	28.92	*trans*-Caryophyllene oxide	220.35	1992	1993	0.53	-	0.55
97.	29.06	Eicosane	282.54	2000	2000	0.30	-	0.93
98.	29.15	Methyl eugenol	178.22	2001	2003	0.19	-	1.03
99.	29.35	Methyl tetradecanoate	242.39	2012	2014	0.16	-	0.23
100.	29.55	Cinnamaldehyde	132.15	2022	2024	0.22	1.12	1.05
101.	29.92	Pentadecanal	226.39	2041	2042	0.04	0.25	0.09
102.	30.17	Octanoic acid	144.21	2051	2052	0.79	0.68	0.48
103.	30.55	Viridiflorol	222.36	2080	2081	-	0.57	0.64
104.	31.35	(+) Spathulenol	220.35	2121	2121	1.18	0.73	1.28
105.	31.4	Hexahydrofarnesyl acetone	268.47	2130	2131	1.51	3.63	1.02
106.	31.6	2-Hydroxy-4-methoxy-benzaldehyde	152.14	2133	2135	-	0.25	-
107.	31.84	α-Bisabolol	222.36	2178	2179	0.18	0.51	0.86
108.	32.01	Eugenol *	164.20	2185	2186	0.17	0.36	2.05
109.	32.19	Nonanoic acid	158.23	2192	2192	0.39	0.69	0.93
110.	32.51	Thymol *	150.21	2197	2198	1.16	0.60	2.74
111.	33.04	Carvacrol *	150.21	2205	2206	0.70	0.57	1.88
112.	33.11	Methyl hexadecanoate	270.45	2210	2210	-	0.16	0.45
113.	33.21	Elemicin	208.25	2213	2215	0.33	0.19	0.74
114.	33.28	Butyl myristate	284.47	2227	2229	0.09	1.90	0.54
115.	33.72	Ethyl hexadecanoate	284.47	2240	2241	-	0.47	0.49
116.	34.11	Decanoic acid	172.26	2262	2264	0.16	-	1.15
117.	34.67	*n*-Tricosane	324.62	2300	2300	0.18	0.49	0.51
118.	35.11	Dihydroactinidiolide	180.24	2321	2324	-	-	2.27
119.	35.41	Octadecanal	268.47	2340	2343	9.37	0.23	0.48
120.	35.99	4-Vinylphenol	120.14	2378	2379	0.38	0.99	0.95
121.	36.13	Isoelemicin	208.25	2389	2390	-	-	0.58
122.	36.33	*n*-Tetracosane	338.65	2394	2396	0.22	0.29	1.29
123.	36.91	Butyl hexadecanoate	312.53	2416	2419	0.16	0.29	0.26
124.	37.69	Dodecanoic acid	200.31	2450	2451	0.16	-	0.54
125.	37.84	9,12-Octadecadienoic acid, methyl ester	2194.47	2457	2459	0.14	0.41	-
126.	38.03	*n*-Pentacosane	352.68	2465	2469	0.19	0.72	0.82
127.	38.8	Vanillin	152.14	2541	2545	-	0.49	0.22
128.	38.92	9,12,15-Octadecatrienoic acid, methyl ester	292.45	2547	2550	0.31	3.42	0.10
129.	39.44	1-Octadecanol	270.49	2580	2581	-	1.86	0.11
130.	39.65	*n*-Hexacosane	366.70	2595	2597	-	0.33	1.02
131.	40.3	n-Butyl octadecanoate	340.58	2654	2655	-	4.92	-
132.	41.03	Tetradecanoic acid	228.37	2698	2698	0.87	0.86	2.05
133.	42.6	Pentadecanoic acid	242.39	2819	2819	0.06	0.38	0.40
134.	42.67	*n*-Octacosane	394.76	2826	2828	0.07	0.29	0.55
135.	44.17	n-Hexadecanoic acid	256.42	2896	2899	1.39	3.16	4.68
136.	45.28	9-Hexadecenoic acid	254.40	2954	2957	0.06	-	0.29
137.	45.5	*n*-Triacontane	422.81	3000	3000	-	4.25	0.22
138.	46.35	Squalene	410.71	3055	3058	-	-	0.10
139.	46.86	*n*-Hentriacontane	436.83	3102	3100	0.10	0.18	0.34
140.	47.02	Octadecanoic acid	284.47	3103	3104	0.05	-	0.22
141.	47.42	9-Octadecenoic acid	282.46	3153	3157	0.10	0.20	0.42
142.	48.14	9,12-Octadecadienoic acid	280.44	3165	3168	0.11	-	0.65
143.	49.05	9,12,15-Octadecatrienoic acid	278.42	3192	3193	0.23	-	1.30
		Total identified compounds %				88.63	70.34	76.78

All the compounds were identified based on the compounds’ mass spectral data and retention indices compared with those of the NIST Mass Spectral Library (December 2011), the Wiley Registry of Mass Spectral Data, 8th edition, and the compounds with * were identified by comparing with many authentic standards. The content (%) was calculated using the normalization method based on the GC-MS data. The presented data are the averages of three independent populations of each species from the same collection area, The standard deviations did not exceed 4% in all identified compounds.

**Table 2:** Antioxidant activities of the volatile oils of *Stachys* species according to the 2,2-diphenyl-1-picryl-hydrazyl-hydrate (DPPH), 2,2-azino bis (3-ethylbenzothiazoline-6-sulphonic acid) (ABTS), cupric reducing antioxidant capacity (CUPRAC), ferric reducing power (FRAP), chelating and phosphomolybdenum (PBD) assays.

Samples	DPPH (mg TE/g oil)	ABTS (mg TE/g oil)	CUPRAC (mg TE/g oil)	FRAP (mg TE/g oil)	Chelating (mg EDTAE/g oil)	PBD (mmol TE/g oil)
*S. byzantina*	Na	13.64 ± 0.02 ^c^	33.17 ± 0.88 ^c^	18.98 ± 0.36 ^c^	12.32 ± 1.76 ^c^	1.45 ± 0.10 ^c^
*S. hissarica*	13.04 ± 0.97	226.48 ± 1.75 ^a^	162.62 ± 1.15 ^b^	109.55 ± 3.24 ^a^	24.30 ± 1.50 ^b^	5.69 ± 0.21 ^b^
*S. betoniciflora*	Na	128.54 ± 1.32 ^b^	174.94 ± 0.20 ^a^	56.94 ± 0.75 ^b^	60.11 ± 0.36 ^a^	28.24 ± 1.00 ^a^

Values are reported as means ± S.D. of three parallel measurements. TE: Trolox equivalent; EDTAE: EDTA equivalent; Na: not active. Different letters ^(a–c)^ in same column indicate significant differences in the tested essential oils.

**Table 3:** Enzyme inhibitory effects of the volatile oils of *Stachys* species.

Samples	AChE Inhibition (mg GALAE/g oil)	BChE Inhibition (mg GALAE/g oil)	Tyrosinase Inhibition (mg KAE/g oil)	Amylase Inhibition (mmol ACAE/g oil)	Glucosidase Inhibition (mmol ACAE/g oil)
*S. byzantina*	5.64 ± 0.04 ^a^	11.18 ± 0.19 ^bc^	101.07 ± 0.60 ^a^	0.59 ± 0.01 ^c^	24.11 ± 0.06 ^c^
*S. hissarica*	5.05 ± 0.01 ^b^	11.82 ± 0.20 ^b^	92.26 ± 2.48 ^b^	0.68 ± 0.02 ^b^	24.56 ± 0.10 ^b^
*S. betoniciflora*	4.09 ± 0.01 ^c^	13.81 ± 0.80 ^a^	75.77 ± 2.24 ^c^	0.76 ± 0.02 ^a^	24.89 ± 0.03 ^a^

Values are reported as means ± S.D. of three parallel measurements. GALAE: Galantamine equivalent; KAE: Kojic acid equivalent; ACAE: Acarbose equivalent; Na: not active. Different letters ^(a–c)^ in same column indicate significant differences in the tested essential oils.

## Data Availability

Data are available upon request from the first author.
